# Rifampicin Pulmospheres for Pulmonary Delivery

**Published:** 2009

**Authors:** M. A. Morde, A. N. Bajaj, R. S. Bhanushali

**Affiliations:** C. U. Shah College of Pharmacy, S. N. D. T. Women's University, Juhu campus, Santacruz (west), Mumbai-400 049, India

**Keywords:** Rifampicin pulmospehres, dry powder inhalations, inhalable lactose, entrapment efficiency, rotahaler

## Abstract

Dry powder inhalation formulations of rifampicin were prepared. Spray drying was used to prepare pulmospheres and their physicochemical characteristics were evaluated. Spray dried pulmospheres containing rifampicin were mixed with inhalable lactose for preparing dry powder inhalation formulations. These formulations were further characterized to evaluate the feasibility of developing effective treatments for pulmonary tuberculosis.

Tuberculosis is most commonly caused by the deposition of bacteria, *Mycobacterium tuberculosis* in the lungs. Pulmonary *tuberculosis* is characterized by alveolar macrophages containing large number of bacilli that are typically about 5 μm in length, facilitating their entry into the lower airways. Antitubercular drug delivery systems can be administered by the pulmonary route to avoid frequent dosing. It could be helpful in direct drug delivery to the lungs, drug targeting to alveolar macrophages, reducing systemic toxicity of the drug. Biodegradable polymers like PLGA are capable of sustained drug release over days to several weeks. Therefore in present study, an attempt was made to develop dry powder inhalation (DPI) formulations of rifampicin (RIF) for pulmonary delivery. DPI formulations of spray dried RIF and of RIF pulmospheres with Poly(DL-lactide-co-glycolide) polymer (75:25) have been developed

## MATERIALS AND METHODS

Rifampicin (Lupin Ltd., Mumbai, India); Pharmatose 325 (DMV international) and Lactohale (Borculo) were generous gift samples. Biodegradable polymer PLGA (75:25) with viscosity of 0.19 dl/g in chloroform as reported by the manufacturer was procured from Birmingham polymers.

### Development of conventional spray dried RIF:

**Fig. 1:**
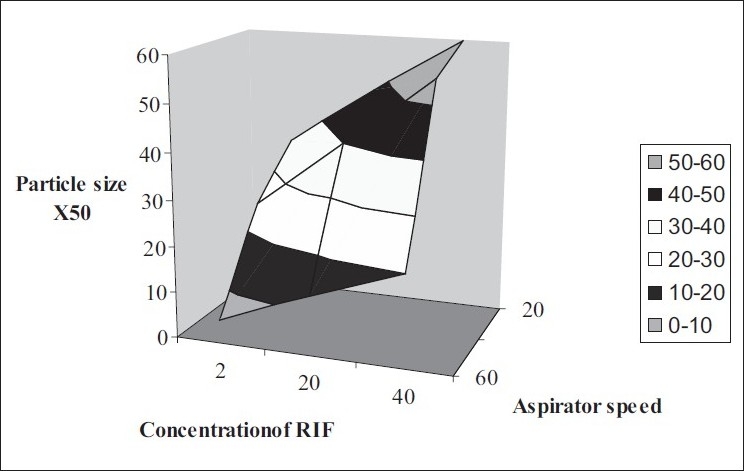
Factorial design for optimization of process parameters Effect of aspirator speed and concentration of drug in solution on particle size X50. The particle sizes were (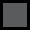) 0-10, (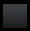) 10-20, (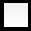) 20-30, (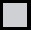) 30-40, (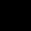) 40-50 and (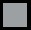) 50-60.

RIF solutions of concentration of 2-40 mg/ml were prepared in dichloromethane and spray dried using Labultima Mini Spray Dryer. Process parameters were optimized using 2^2^ factorial design. Effect of process parameters on particle size was studied (fig. 1).

### Preparation and characterization of RIF-loaded pulmospheres:

Spray drying technology was used to prepare pulmospheres. The solution of polymer containing drug was spray dried to generate polymer-coated particles. Developed pulmospheres were evaluated for physiochemical properties like particle size, surface characteristics, % drug loading and entrapment efficiency. DSC studies were performed.

### Formulation development of DPI:

**Fig. 2:**
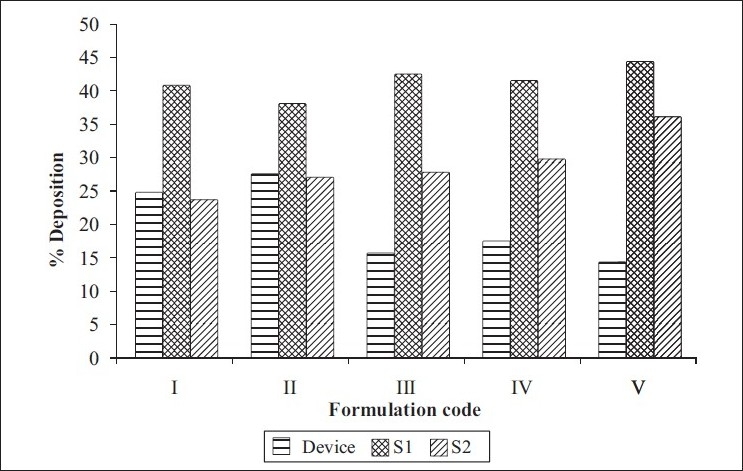
*In vitro* pulmonary deposition patterns of spray dried RIF *In vitro* pulmonary deposition patterns of spray dried RIF conventional DPI formulation. (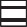) device, (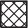) S1 and (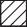) S2

**Fig. 3:**
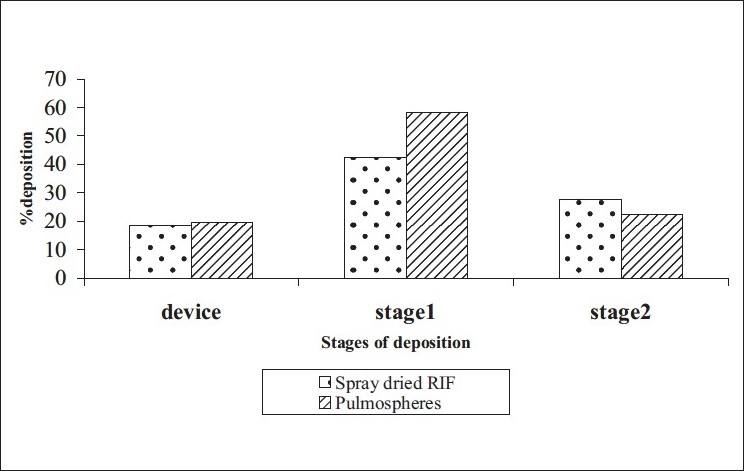
Comparison of respirable fractions Comparison of respirable fraction of conventional DPI formulation and DPI with pulmospheres. (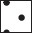) spray dried RIF and (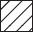) pulmospheres

Spray dried RIF/pulmospheres of RIF were mixed with inhalable lactose in varying ratios and developed DPI formulations were characterised (Table 1). The optimum ratio of coarse lactose to fine lactose that gives maximum respirable fraction was selected (fig. 2). *In vitro* aerolisation behaviour of the formulations was evaluated using a Rotahaler, and the performance was characterised based on uniformity of emitted dose and aerodynamic particle-size distribution (respirable fraction (RF), as a percentage of nominal dose (RFN) and emitted dose (RFE). DPI formulations of spray dried RIF and PLGA-based RIF Pulmospheres were compared in terms of respirable fraction delivered (fig. 3).

**TABLE 1:** QUALITY CONTROL TESTS PERFORMED ON CONVENTIONAL RIF DPI FORMULATIONS

Batch code	Assay (%w/w)	bulk density (gm/cc)	Moisture content (% w/w)	% deposition in stage ii
D1	82	0.4	0.2	26.49
D2	85	0.32	0.38	29.15
D3	90	0.41	0.5	32.3
D4	95	0.38	0.8	33.4
D5	98	0.33	0.5	36

### *In vitro* release studies:

Release studies were performed by dialysis method. The diffusion cell was kept at 37° with continuous stirring at 100 rpm and RIF was analyzed spectrophotometrically at 475 nm. Coefficient of correlation from plots of Q vs. t, (cumulative % drug release vs. time), log of percent drug retained vs. t and Q vs. square root of t were calculated to determine drug release.

## RESULTS AND DISCUSSION

**Fig. 4:**
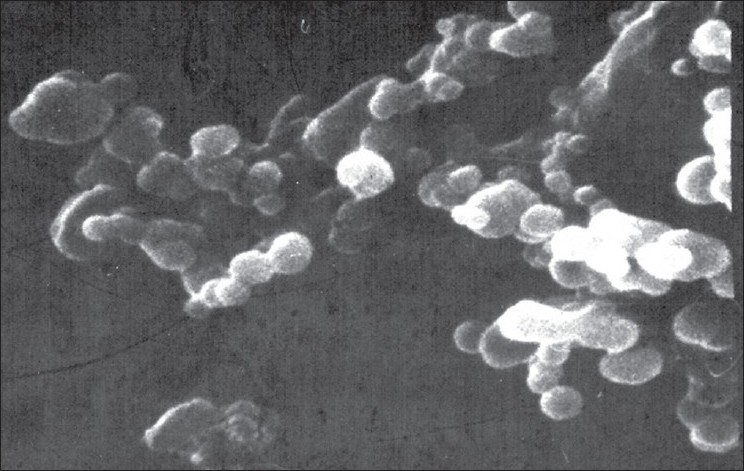
SEM micrograph of RIF pulmospheres

**Fig. 5:**
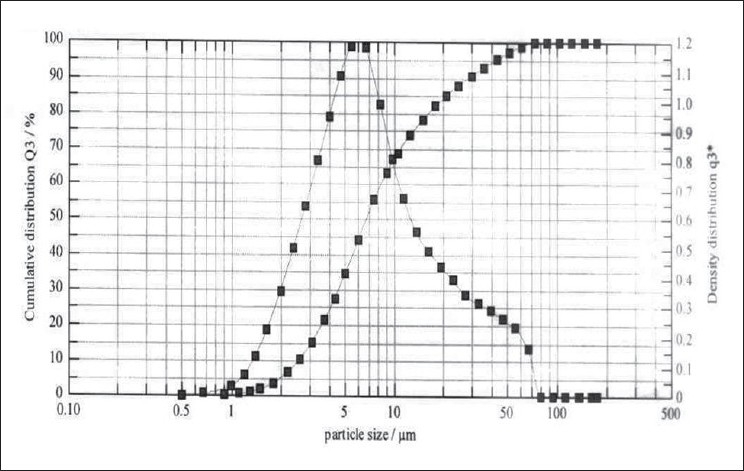
Particle size distribution of RIF Pulmospheres

**Fig. 6:**
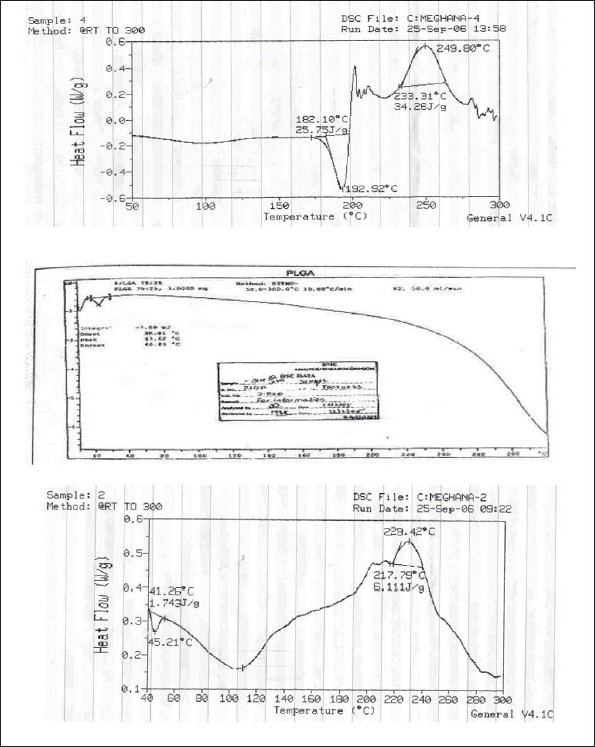
DSC spectra of developed formulation (a) RIF (b) PLGA (c) Pulmospheres

**Fig. 7:**
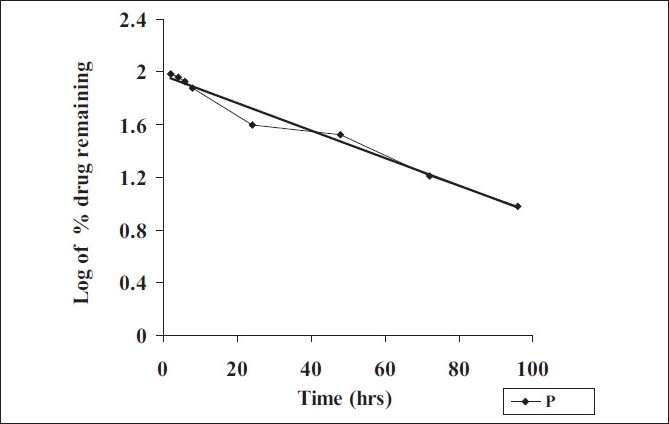
*In vitro* release profile of RIF pulmospheres

Optimum process parameters for spray dried RIF were inlet temperature (55°), aspirator rate (60 l/min), feed rate (10 ml/min) and optimum concentration was 2 mg/ml (fig. 1). DPI formulations were developed using various grades of inhalable lactose like pharmatose and Lactohale in various combinations. The effect of particle size of excipients on respirable fraction of RIF was assessed (Table 1, fig. 2). Pulmospheres of RIF were prepared with PLGA (1:1, drug:polymer ratio) with 96% v/v entrapment efficiency. Developed PLGA porous pulmospheres were characterized by SEM analysis (fig. 4) and for particle size (fig. 5), % drug entrapment and %FPF (Table 2). DSC studies confirmed no interaction between drug and polymer (fig. 6). Regression coefficients (near to 1) for zero order, first order and Higuchi's model equations confirmed release by first order (R^2^=0.97887) (fig. 7). Spray dried RIF and Pulmospheres exhibited excellent flow and dispersion from passive DPIs while pulmospheres released for longer period of time. *In vitro* characterization has predicted highly efficient lung delivery of RIF for treatment of pulmonary tuberculosis.

**TABLE 2:** RESULTS OF OPTIMISED DPI FORMULATIONS PREPARED BY SPRAY DRYING TECHNOLOGY

Parameters	Observations	
	
	Spray Dried RIF	Pulmospheres
Appearance	Spherical	Hollow porous
Pariticle size	1-10μm	1-10μm
%Yield	48	45.6
%moisture content	0.5	0.75
% drug entrapment	-	96
%FPF	36	32
